# Seasonal Developing Xylem Transcriptome Analysis of *Pinus densiflora* Unveils Novel Insights for Compression Wood Formation

**DOI:** 10.3390/genes14091698

**Published:** 2023-08-26

**Authors:** Thi Thu Tram Nguyen, Min-Ha Kim, Eung-Jun Park, Hyoshin Lee, Jae-Heung Ko

**Affiliations:** 1 Department of Plant & Environmental New Resources, Kyung Hee University, Yongin 17104, Republic of Korea; nguyenthutram1991@khu.ac.kr (T.T.T.N.); minha123@khu.ac.kr (M.-H.K.); 2 Forest Bioresources Department, National Institute of Forest Science, Suwon 16631, Republic of Korea; pahkej@korea.kr (E.-J.P.); hyoshinlee@korea.kr (H.L.)

**Keywords:** conifer, gymnosperm, compression wood, season, *Pinus densiflora*, lignin biosynthesis, wood formation

## Abstract

Wood is the most important renewable resource not only for numerous practical utilizations but also for mitigating the global climate crisis by sequestering atmospheric carbon dioxide. The compressed wood (CW) of gymnosperms, such as conifers, plays a pivotal role in determining the structure of the tree through the reorientation of stems displaced by environmental forces and is characterized by a high content of lignin. Despite extensive studies on many genes involved in wood formation, the molecular mechanisms underlying seasonal and, particularly, CW formation remain unclear. This study examined the seasonal dynamics of two wood tissue types in *Pinus densiflora*: CW and opposite wood (OW). RNA sequencing of developing xylem for two consecutive years revealed comprehensive transcriptome changes and unique differences in CW and OW across seasons. During growth periods, such as spring and summer, we identified 2255 transcripts with differential expression in CW, with an upregulation in lignin biosynthesis genes and significant downregulation in stress response genes. Notably, among the laccases critical for monolignol polymerization, PdeLAC17 was found to be specifically expressed in CW, suggesting its vital role in CW formation. PdeERF4, an ERF transcription factor preferentially expressed in CW, seems to regulate PdeLAC17 activity. This research provides an initial insight into the transcriptional regulation of seasonal CW development in *P. densiflora*, forming a foundation for future studies to enhance our comprehension of wood formation in gymnosperms.

## 1. Introduction

Lignocellulose, derived from woody plants, serves as a valuable source for biofuel production [1,2]. Wood is composed of a secondary cell wall (SCW) enriched with cellulose, hemicellulose, and lignin. To improve the properties of woody biomass, it requires a comprehensive understanding of the transcriptional regulation of the biosynthesis of these components. The process of wood formation (also known as xylogenesis) involves cell division and expansion of cambial initials, xylem cell differentiation, SCW deposition, and programmed cell death (PCD) [3,4]. This coordinated control appears to be the result of a complex, multilayer transcriptional regulatory network [5,6].

Distinct weather conditions (e.g., day length, air temperature, and precipitation) mark seasons, significantly influencing the genetic regulations in plants, particularly woody trees like gymnosperms. During spring and summer, these trees actively grow, stimulating wood formation. Conversely, during fall and winter, they enter dormancy, triggering defense and stress response mechanisms [7,8]. Previous research underscores temperature as a crucial determinant of wood formation onset in gymnosperms in the Northern hemisphere. Notably, extreme conditions such as heat, drought, and low spring precipitation can delay the growth season [9,10]. The mechanisms terminating wood formation remain largely unexplored. Factors like low temperatures, short photoperiods, or drought stress may be involved, depending on the geographical location [8,9,10].

A critical feature of xylem cells is the formation of reaction wood, distinct in angiosperms (tension wood, TW) and gymnosperms (compression wood, CW). These adaptations enable trees to resist environmental forces [11,12]. In gymnosperms, stem inclination triggers CW formation in the lower side with abundant lignin, through the induction of auxin and ethylene, and the reduction in jasmonic acid (JA) [13]. In contrast, TW formation in angiosperms has a high cellulose content and is formed in the upper side of an inclination of a stem [12]. Recently, many studies have been conducted on TW formation in various angiosperm species, including *Catalpa bungee*, *Populus trichocarpa*, *Hevea brasiliensis*, and *Betula luminifera*, to understand the underlying molecular mechanisms and identify essential genes [14,15,16,17,18]. Previously, Sato et al. [19] reported transcriptome analysis of reaction wood formation from *Chamaecyparis obtusa*. However, research on CW formation in gymnosperms, especially conifers, is still limited [19,20,21].

Gymnosperm wood is characterized by high lignin content (25–35%), an HG-type of lignin with more guaiacyl (G) units but a small portion of p-hydroxyphenyl (H) units., whereas angiosperm wood has a lignin content of 15–28%, with a GS-lignin having different proportions of syringyl (S) units [22,23]. It has recently been shown that laccase, responsible for monolignol polymerization, is crucial for lignin biosynthesis in compression wood [22,24]. Lignin-derived fuels have gained popularity in recent years as a technology to produce biofuels from biomass with the potential to reduce dependence on fossil fuels and greenhouse gas emissions [25]. Thus, CW of gymnosperms can be an excellent source of lignin for biofuel production.

Genomic analyses of conifers have been challenging due to their enormous genome sizes and high heterozygosity. The genome size of the Norway spruce (*Picea abies*), for instance, is 20 Gb, seven times larger than the human genome [26]. Conifers have recently been able to undergo genome-wide investigation via EST sequencing [27], whole-genome sequencing, and RNA sequencing (RNA-Seq) [28]. The Norway spruce [26], White spruce (*Picea glauca*, [29]), Loblolly pine (*Pinus taeda*, [30]), and Sugar pine (*Pinus lambertiana*, [31]) are some conifer species that have draft genome assemblies available. By RNA sequencing and PacBio SMRT iso-sequencing, a comprehensive transcriptome profile of Korean red pine (*Pinus densiflora*) was reported recently, which identified key genes involved in the biosynthesis of cellulose, hemicellulose, and lignin [4]. *P. densiflora* has a native range that includes the Korean Peninsula, China, Japan, and East Russia and is the most abundant tree species in South Korea, covering up about 87% of coniferous forests [32]. *P. densiflora* is an important source of wood in South Korea [33], with the trunks containing many resins that can be used to make rosin and turpentine [34] and the leaves being used to make essential oils [32]. In this study, we examined the transcriptional dynamics of CW formation in P. densiflora across different seasons. The findings will contribute a comprehensive overview of transcriptional regulation of CW formation, providing a platform for future investigations into gymnosperm wood formation.

## 2. Materials and Methods

### 2.1. Wood-Forming Tissue Sampling and RNA Sequencing

**Figure 1:**
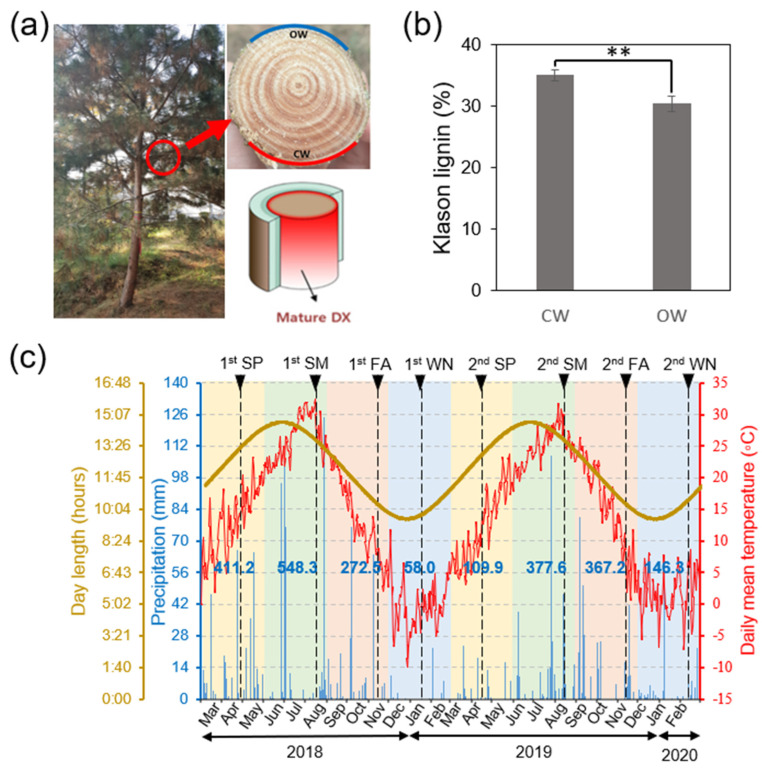
Strategy for wood tissue sampling from *P. densiflora* used in this study. (**a**) Sampling of MDX (Mature Developing Xylem) tissues from branches of P. densiflora grown in nursery. After de-barking, the upper part (blue line) was collected as opposite wood (OW) with the lower part as compression wood (CW) (red line) (see, Methods). (**b**) Quantification of Klason lignin from CW and OW. Error bars indicate SD (n = 3). Student *t*-test, ** (*p* < 0.01). (**c**) Seasonal sampling and weather condition. Sampling dates of two year were pointed by black arrow heads on top. Day length (yellow line), precipitation (blue lines), and average temperature (red line) from March 2018 to February 2020 were shown in left and right axis, respectively. Total amounts of precipitation (mm) in each season were shown in the middle of the graph. The weather data were obtained from Korea Astronomy and Space Science Institute (KASI; “https://astro.kasi.re.kr (accessed on 20 August 2023)”) and Korea Meteorological Administration (KMA; https://www.weather.go.kr (accessed on 20 August 2023)). CW (Compression Wood), OW (Opposite Wood), SP (Spring), SM (Summer), FA (Fall), and WN (Winter).

Samples were collected from 14-year-old Korean red pine (*P. densiflora*) grown in the Korea Forest Research Institute (KFRI, Suwon, Republic of Korea, 37°15′ N, 126°57′ E), between 2018 and 2020 (approximately 9.5 m in height and 15.5 cm in diameter at breast (DBH)). In branches of *P. densiflora*, the lower side with high lignin was classified as CW and the upper side as OW. Developing xylem (DX) of CW and OW was collected during four seasons: spring (SP), summer (SM), fall (FA), and winter (WN) in the indicated time by scraping the surface of debarked branches, respectively (Figure 1a, Supplementary Table S1). Each sample was collected from a branch at DBH of the tree to ensure the same developmental stage. Details about temperature, day length, and precipitation during the sampling period can be found in Figure 1c, with information sourced from the Korea Astronomy and Space Science Institute (KASI; “https://astro.kasi.re.kr (accessed on 20 August 2023)”) and the Korea Meteorological Administration (KMA; “https://www.weather.go.kr (accessed on 20 August 2023)”). The temperature and day length on sampling day were presented in Supplementary Table S1.

Total RNA was isolated from each tissue (pooled from three trees) using RNAprep Pure Plant Plus kit (Tiangen, Beijing, China), and RNA quality was determined by a 2100 Expert Bioanalyzer (Agilent, Santa Clara, CA, USA). Preparation of cDNA library was constructed according to the Truseq Stranded mRNA Preparation Kit (Illumina, San Diego, CA, USA). Paired-end sequencing was performed on an Illumina NovaSeq 6000 platform with a read length 2 × 101 bp. The resulting RNA sequencing data were deposited in NCBI under BioProject accession PRJNA789345, and the summary of the results was performed in Supplementary Table S2.

### 2.2. Transcript Assembly, Abundance Estimation, and Annotation

Transcriptome analysis was performed following the published method [4]. In brief, after RNA sequencing, PRINSEQ-Lite 0.20.4 (“http://prinseq.sourceforge.net/ (accessed on 20 August 2023)”) was used for cleaning the paired-end raw reads (Phred quality above 20, and the minimum length is 50 nucleotides). Then, the cleaned reads were used to generate a reference transcriptome for *de novo* transcriptome assembly, using a script in Trinity v2.6.6 [35,36] with a default parameter. Clustering of transcripts was performed using CD-HIT-EST v4.6 with a default parameter [37,38]. Bowtie v. 1.2.2 was used for mapping the cleaned reads of each library to the de novo assembled reference transcriptome [39]. Transcript abundance (i.e., read count) was determined by RSEM v. 1.3.0 [40], and the raw counts were normalized as the trimmed mean of M-values (TMM)-normalized transcripts per million (TPM). De novo assembled reference transcriptome genes were annotated with *A. thaliana* (Athaliana_167_TAIR10) and *P. trichocarpa* (Ptrichocarpa_210_v3) protein datasets from Phytozome V12 (https://phytozome.jgi.doe.gov/pz/portal.html (accessed on 20 August 2023)) using BLASTX (e-value 1 × 10^−5^; max_target_seqs 1). Principal component analysis was performed using the platform iDEP [41].

### 2.3. Differentially Expressed Gene and Gene Ontology Analysis

The differentially expressed genes (DEGs) were identified using log_2_ fold change (log_2_FC) values of TPM, with a threshold of 1.5 or −1.5. The growing season up-regulated genes (in CW or OW) had higher expressions during the growing season than dormancy, with a log_2_FC of SP or SM versus FA ≥ 1.5. On the other hand, the growing season down-regulated genes had lower expressions during the growing season than dormancy, with a log_2_FC of SP or SM versus FA ≤ −1.5. In the growing season (SP or SM), CW up-regulated genes had higher expressions in CW than OW, with a log_2_FC of CW versus OW ≥ 1.5. The CW down-regulated genes had lower expressions in CW than OW, with a log_2_FC of CW versus OW ≤ −1.5. The threshold TPM of 1.0 was used to identify whether genes were significantly expressed.

Gene ontology (GO) enrichment analysis was performed using DAVID (“https://david.ncifcrf.gov/tools.jsp (accessed on 20 August 2023)”, [42]). The biological process (BP) GO was found to be substantially enriched at the threshold of *p*-value as 0.03. Following the removal of redundancy terms, the top specific terms were reported in Figures 3 and 6, indicating the performance of negative logarithmic adjusted *p*-value of enrichment.

### 2.4. Quantitative Real Time PCR (qRT-PCR) and Semi-Quantitative Reverse Transcription PCR (RT-PCR)

One microgram of total RNA was reverse transcribed to produce first-strand cDNA using Superscript III reverse transcriptase (Invitrogen, Carlsbad, CA, USA) in 20 µL reaction volume. The gene expression pattern was confirmed by quantitative real-time PCR (qRT-PCR) and semi-quantitative PCR (RT-PCR) of both two sets of samples. The qRT-PCR was performed using an AriaMx Real-time PCR system (Agilent, Houston, TX, USA) with Brilliant III Ultra-Fast SYBR Green QRT-PCR Master Mix (Agilent, Houston, TX, USA). Subsequent RT-PCR was carried out using 1 µL of the reaction product as the template. *PdeUBC11* gene was used as the quantitative control [4]. All primer sequences were designed using the Primer3 program (“http://primer3.ut.ee (accessed on 20 August 2023)”) (Supplementary Table S6).

### 2.5. Klason Lignin Quantification

Klason lignin (i.e., acid-insoluble lignin) contents of CW and OW in SM of the first sample set were measured following the reported method [43]. In detail, debarked branch tissues were dried at 65 °C for 1 week and ground to a fine powder. Ground samples (∼100 mg) were placed in glass screw-cap tubes, and 1 mL of 72% (*v*/*v*) sulfuric acid was added followed by complete mixing. The tubes were placed in a water bath set at 45 ± 3 °C and incubated for 90 ± 5 min until all samples were completely hydrolyzed. The acid was diluted to a 4% concentration by adding 28 mL of deionized water. Samples were mixed by inversion several times to eliminate phase separation. Samples were autoclaved for 1 h at 121 °C and slowly cooled down to room temperature before removing the caps of the tubes. The autoclaved hydrolysis solution was vacuum filtered through pre-weighed filter paper. The filter paper was dried at 65 °C to obtain acid-insoluble residue until a constant weight was achieved. The filter paper was allowed to cool down to room temperature and the weight of the filter paper and dry residue was recorded.

### 2.6. Transient Overexpression Analysis by the Agrobacteria-Infiltration Method and Laccase Activity Assays

Full-length coding sequences of *PdeERF4* (DN57342_c0_g1_i2, 813 bp), *PdeMYB106* (DN63531_c0_g3_i10, 985 bp), and *AtMYB46* (AT5G12870.1, 843 bp) were inserted downstream of the 35S promoter in the pB2GW7 vector [44] using the Gateway cloning method (Invitrogen, Carlsbad, CA, USA) to produce the 35S::PdeERF4, 35S::PdeMYB106, and 35S::AtMMYB46 constructs. Vector constructs were transformed into *Agrobacterium tumefaciens* (strain C58) that was then used for agrobacteria infiltration, following the reported method [4]. Agrobacterium cells carrying empty plasmid (pB2GW7) were used as a negative control, whereas the 35S::AtMYB46 construct was used as the positive control for infiltration efficiency. Extracellular protein extraction and in vitro laccase activity assay (ABTS assay) were modified from the method used for Arabidopsis [45]. In brief, 300 mg of tobacco-infiltrated leaf was ground into fine powder in liquid nitrogen, then mixed with 300 µL of protein extraction buffer (25 mM BisTris (pH 7.0), 10% glycerol (*v*/*v*), 200 mM CaCl2, 0.1 mM EDTA, and protease inhibitor cocktail (cOmplete™, Mini Protease Inhibitor Cocktail, Merk, Darmstadt, Germany)), then incubated in ice 5 min. The homogenate was centrifuged in 3000× *g* for 5 min at 8 °C and then at 4 °C for 5 min in 13,000× *g*. Finally, it was centrifuged at 8 °C for 45 min in 15,000× *g*. The supernatant was taken into a new chilled tube after every centrifugation. The crude protein was quantified based on Bradford dye-binding method [46]. Laccase activity was determined by the oxidation of ABTS (3-ethylbenzthiazoline-6-sulfonic acid, Merk, Germany) to generate a stable cationic radical (measure at the absorbance of 420 nm). The reaction mixture (1 mL) contains 900 µL of 1 mM ABTS (diluted in 0.1 M sodium acetate buffer pH 5.0), and 100 µL of crude protein (1.8 µg/µL) was incubated overnight at 25 °C, based on the optimum condition of laccase activity [47]. A total of 500 ng/mL of laccases from *Trametes versicolor* (Merk, Darmstadt, Germany) was used as a positive control for the ABTS assay.

## 3. Results

### 3.1. Analyzing the Seasonal Xylem Development in P. densiflora

We conducted a comprehensive study on the molecular mechanisms governing the formation of compression wood (CW) in conifers throughout different seasons. To accomplish this, we developed a whole-genome transcriptomic profile of developing xylem (DX) from both CW and the opposite wood (OW) in *P. densiflora*. DX samples from CW and OW of branches were collected at specific times during spring (SP), summer (SM), autumn (FA), and winter (WN) over two years (Figure 1a,c). The DX sample from CW, recognized by its larger, darker appearance compared to OW, was gathered from the bottom portion of the branch (Figure 1a). As anticipated, we found that the CW had significantly more lignin content (35.0%) than the OW (30.4%), which aligned with previous findings in *P. taeda* (Figure 1b) [48].

Our samples were collected towards the end of each season, which corresponded to the periods of the growing season (SP), late growing season (SM), mid-dormancy (FA), and late dormancy (WN). Daily average temperatures varied significantly, with the warmest temperatures occurring in summer and the coldest in winter (Figure 1c, Supplementary Table S1). The day length remained similar from spring through summer (around 13 h), before decreasing in autumn and winter (around 10 h) (Figure 1c, Supplementary Table S1). Although patterns in temperature and day length changes were relatively uniform across our two-year study period, precipitation levels varied significantly. The precipitation in SP of the 1st set was much higher (about 3.7 times) than in the 2nd set, and the precipitation in SM of the 1st set was slightly higher (about 1.45 times) than in the 2nd set (Figure 1c).

To evaluate the quality of the tissue samples before performing RNA-seq for transcriptome analysis, we tested several marker genes related to wood formation using semi-quantitative RT-PCR (Figure S1). *PdeNAC2* and *PdeMYB46*, close homologues of Arabidopsis *MYB46* and *VND6*, respectively, were highly expressed during growing season (SP and SM) in both CW and OW and minimally expressed or not at all during the dormant season (FA and WN) (Figure S1). Similarly, SCW-specific cellulose synthases (i.e., *PdeCesA7* and *PdeCesA8*), lignin monomer biosynthetic genes (*Pde4CL*, *PdeC3H*, *PdeCOMT*, *PdeCCoAOMT1*, *PdeCCR*) and lignin polymerization genes (*PdeLAC3*, *PdeLAC10*, *PdeLAC12*) were predominantly expressed in SP and SM in both CW and OW (Figure S1). Some lignin monomer biosynthesis genes (e.g., *PdeC4H* and *PdeCAD*) were also expressed in FA and WN, suggesting their roles in defense mechanisms [14]. Interestingly, *PdeLAC17* was exclusively expressed in CW, not OW, during the growing season.

### 3.2. Building the Seasonal Transcriptomes of the Developing Xylem of P. densiflora

We constructed a transcriptome library using 16 samples, capturing both CW and OW across four seasons, with data collected over two years. From these, we obtained a total of 526,109,063 reads, with more than 95% of reads having a Q30 score or higher and an average quality score of 36.36 (Supplementary Table S2). These reads allowed us to assemble 326,226 contigs, with an N50 value of 1551 bp and an Ex90N50 value of 2288 bp. Using BLASTX, 105,984 contigs (32.48%) and 105,413 contigs (32.31%) were matched to protein sequences of *A. thaliana* and *P. trochocarpa*, respectively.

**Figure 2:**
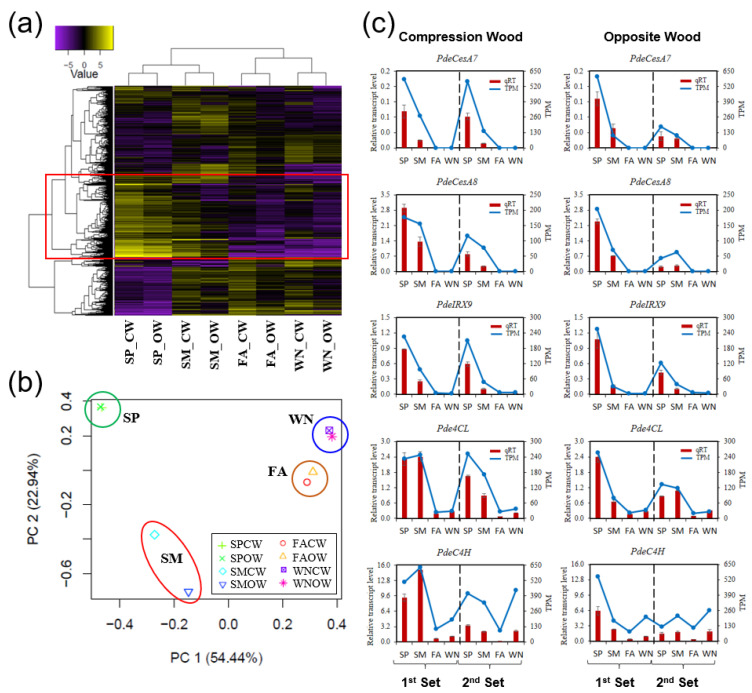
Seasonal wood tissue-specific transcriptome analysis of *P. densiflora*. (**a**) Sample correlations are illustrated using a heat map. SP, spring; SM, summer; FA, fall; WN, winter. CW, compression wood; and OW, opposite wood. Average values of two-set biological replicates (log2 fold change value with *p*-value, 0.005) were used in the Trinity package (analyze_diff_expr.pl). Clusters up-regulated in SP-SM are marked in red box. (**b**) Principal component analysis (PCA) plot was generated using the same dataset described in (**a**) in iDEP.91 (http://bioinformatics.sdstate.edu/idep/ (accessed on 20 August 2023)) with default parameters. (**c**) Validation of the RNA-seq data by marker genes of secondary cell wall formation. In each plot, blue line (right *Y*-axis) represents TPM (Transcript Per Million) values of the indicated gene from RNA-seq data while red bars (left *Y*-axis) show the results of independent qRT-PCR experiments. Error bars indicate SD (n = 3).

We evaluated the transcriptional interrelationships of eight tissue libraries through a hierarchical clustering analysis in the Trinity package [36] using the average values of two-year biological replicates (Figure 2a). As expected, it showed distinct groupings for the growing (SP and SM) and dormant seasons (FA and WN) (Figure 2a). A Principal Component Analysis (PCA) showed a similar grouping, with some interesting observations (Figure 2b).

The transcriptomes of FA and WN, both dormant seasons, exhibited strong similarities, as evidenced by their proximity in Figure 2b. However, despite both SP and SM being classified as growing seasons, their transcriptomes displayed notable differences, with a clear separation. Particularly intriguing is the clear distinction between CW and OW in the summer; in contrast, their transcriptomes appeared very similar during the other seasons. This suggested unique transcriptomic characteristics in CW and OW specifically during the summer.

To validate our RNA-seq data, we analyzed the expression patterns of several well-known marker genes involved in secondary wall formation (e.g., *PdeCesA7*, *PdeCesA8*, *PdeIRX9*, *Pde4CL*, and *PdeC4H*) using qRT-PCR in both two sampling sets. All of them had similar expression patterns with TPM values from RNA seq data, which were highly expressed in SP-SM but decreased in FA-WN (Figure 2c). These results confirmed our RNA-seq data as sufficiently reliable for further analyses (Figure 2c).

### 3.3. Coordinated Gene Expression Shapes Seasonal CW and OW Formation

In the growing season (SP and SM), we identified 9367 up-regulated and 8174 down-regulated differentially expressed genes (DEGs), with more DEGs in the spring than in the summer (Supplementary Figure S2). GO analysis suggested the up-regulated genes played roles in xylogenesis (e.g., cell division, cell differentiation, pectin, lignin, cellulose, xyloglucan, xylan biosynthesis, etc.) and hormone regulation (e.g., auxin, cytokinin, and brassinosteroid), whereas down-regulated genes seemed to be involved in stress response (e.g., defense, salt stress, jasmonic acid (JA), ethylene, and water deprivation response) (Supplementary Figure S2c).

**Figure 3:**
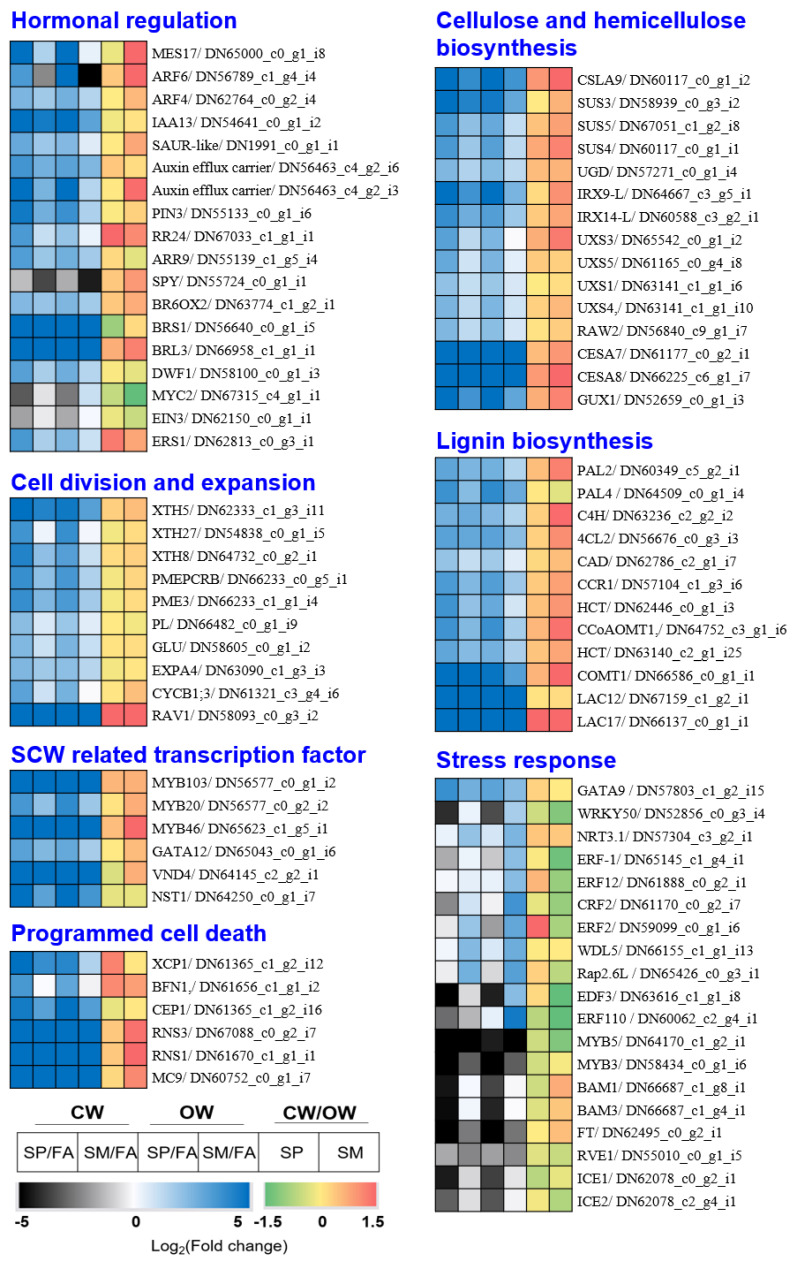
Seasonal CW and OW formation involves the coordinated expression of a diverse gene repertoire. Heatmaps of each gene repertoire (i.e., hormonal regulation, cell division and expansion, SCW-related transcription factor, programmed cell death, cellulose and hemicellulose biosynthesis, lignin biosynthesis, and stress response) show the up- or down-regulation of each gene expression in the growing season (SP and SM) compared to dormant season (FA) (represented by blue to black color bars) and the change in gene expression in CW versus OW in the growing season (by red to green color bars). Detailed gene expressions can be found in the Supplementary Table S2, and the color scale bars are located at the bottom left. CW (Compression Wood), OW (Opposite Wood), SP (Spring), SM (Summer), and FA (Fall).

To investigate the genes associated with seasonal changes in CW and OW formation, we visualized gene expression using heatmaps across seven repertoires, including hormonal regulation, cell division and expansion, SCW-related transcription factor, programmed cell death, cellulose and hemicellulose biosynthesis, lignin biosynthesis, and stress response (Figure 3). We observed that some genes were strongly upregulated in spring and then downregulated in summer, whereas others showed a steady increase from spring to summer (Figure 3). For example, genes associated with auxin and cytokinin signaling (e.g., *MES17*, *ARF4/6*, *IAA13*, *SAUR-like*, *auxin efflux carrier*, *PIN3*, *RR24*, and *ARR9*) had a significant upregulation in spring but declined in summer, whereas those related to brassinosteroid signaling (e.g., *BR6OX2*, *BRS1*, *BRL3*, and *DWF1*) continuously increased from spring to summer. Interestingly, genes that negatively regulate gibberellin signaling (i.e., *SPY*) and a jasmonic acid signaling gene (i.e., *MYC2*) were less active in both spring and summer.

Most wood formation-related genes (e.g., cell division and expansion, SCW-related transcriptional factors, SCW biosynthesis, and PCD) were significantly upregulated in spring, with many stress response genes downregulated in CW during both spring and summer. Certain genes were found to have a higher expression in summer (e.g., *WRKY50*, *NRT3.1*, *ERF-1*, etc.) (Figure 3, Supplementary Table S3). The CW/OW fold change was similar in SP in most of the wood formation genes, whereas some genes were higher in SM (e.g., *MES17*, *ARF6*, *Auxin efflux carrier*, *MYB46*, *CSLA09*, *UXS3*, *CESA8*, *PAL2*, etc.) (Figure 3, Supplementary Table S3).

Taken together, our research reveals that the expression of genes involved in wood formation, hormone regulation, and stress response varies seasonally, suggesting a complex interplay of molecular mechanisms driving wood formation in *P. densiflora*.

### 3.4. Evaluating Seasonal Variations in Gene Expression Involved in Wood Formation

**Figure 4:**
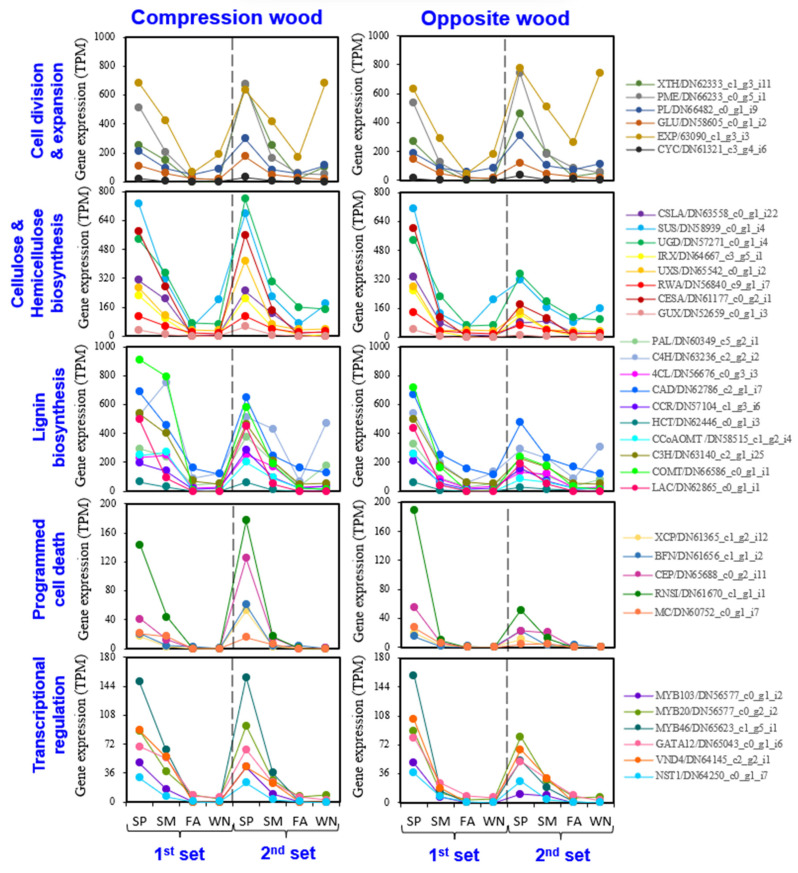
Expressional changes in genes involved in the wood formation (CW and OW) over four seasons in two consecutive years. Expression of genes involved in the wood formation process (i.e., cell division and expansion, cellulose andhemicellulose biosynthesis, lignin biosynthesis, programmed cell death, and transcriptional regulation) were presented. XTH (xyloglucan endotransglycosylases), PME (pectin methylesterase), PL (pectate lyase), GLU (beta-1,3-glucanase), EXP (expansin), CYC (cyclin), CSLA (cellulose synthase-like A), SUS (sucrose synthase), UGD (UDP-Glucose Dehydrogenase), IRX (irregular xylem), UXS (UDP-xylose synthase), RWA (reduced wall acetylation), CESA (cellulose synthase A), GUX (glucuronic acid substitution of xylan), PAL (phenylalanine ammonia-lyase), C4H (cinnamate 4-hydroxylase), 4CL (4-coumarate: coa ligase), CAD (cinnamyl-alcohol dehydrogenase), CCR (cinnamoyl-coa reductase), HCT (hydroxycinnamoyl-coa:shikimate/quinate hydroxycinnamoyltransferase), CCoAOMT (caffeoyl-coenzyme a 3-o-methyltransferase), C3H (coumaric acid 3-hydroxylase), COMT (caffeic acid o-methyltransferase), LAC (laccase), XCP (xylem cysteine protease), BFN (bifunctional nuclease1), CEP (cysteine endopeptidases), RNSI (class I RNAse), NAC (NAM, ATAF, and CUC), MYB (MYB transcription factor), GATA (GATA binding transcription factor), VND (vascular-related NAC-domain), and NST (NAC secondary wall thickening promoting factor). CW (Compression Wood), OW (Opposite Wood), SP (Spring), SM (Summer), FA (Fall), WN (Winter). SP, spring; SM, summer; FA, fall; and WN, winter. TPM, transcripts per million.

Figure 1c shows that there were no significant changes in temperature and day length during the two sequential years of our study, but precipitation varied considerably between seasons, especially in spring. Hence, we evaluated the expression of genes associated with wood formation (including cell division and growth, cellulose and hemicellulose synthesis, lignin biosynthesis, programmed cell death, and transcription regulation) across CW and OW formation for these two years (Figure 4).

Mostly, the genes related to wood formation followed a typical seasonal pattern—high expression during spring and summer and low during dormancy (fall and winter). In CW, gene expression was consistently high during spring of both years (Figure 4). However, in OW, except for genes controlling cell division and expansion, the expression was lower during the spring of the second year, compared to the first (Figure 4). We confirmed these results using additional genes (Supplementary Figure S3).

However, despite these observations, the precise influence of changing precipitation patterns on these gene expression shifts remains unclear and warrants further investigation.

### 3.5. Uncovering Key Genes in the SCW Biosynthesis Crucial for CW Formation in P. densiflora

**Figure 5:**
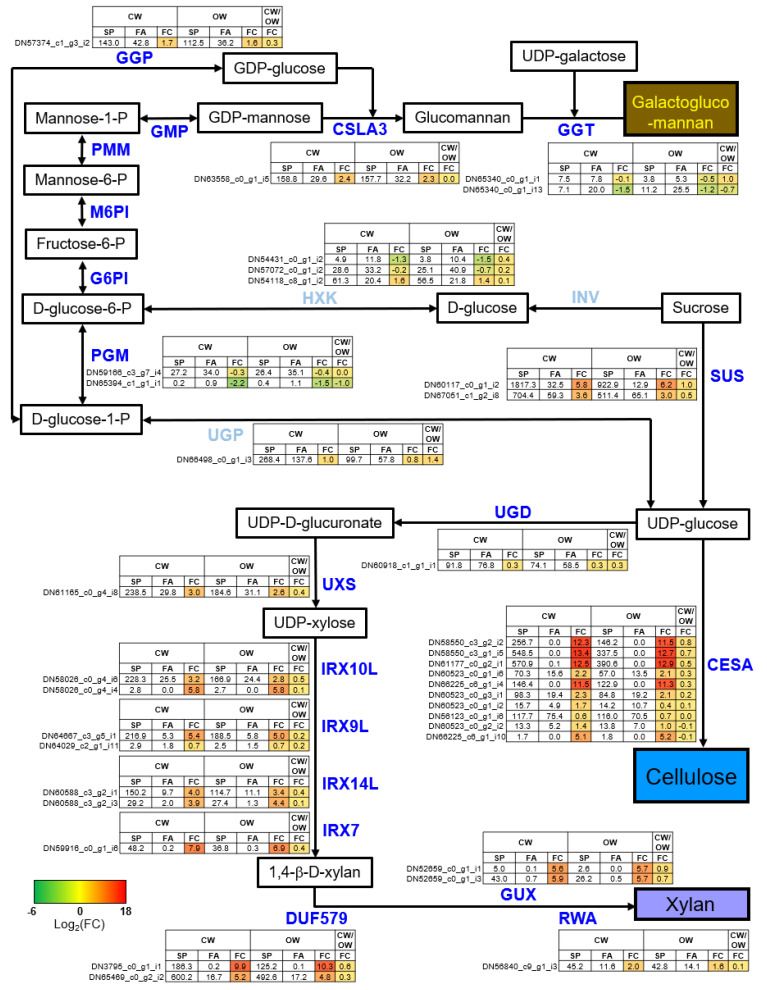
The seasonal transcriptional regulation of genes in cellulose, galactoglucomannan, and xylan biosynthetic pathways for secondary wall formation. Metabolites in each step of the biosynthetic pathway are shown in the box, and all related genes are shown to the side. *SUS* (Sucrose Synthase); *CESA* (Cellulose Synthase A); *UGD* (UDP-Glucose Dehydrogenase); *UGP* (UDP-Glc Pyrophosphorylase); *PGM* (Phosphoglucomutase); *UXS* (UDP-Xylose Synthase); *IRX10L* (Irregular Xylem 10-Like); *IRX9L* (Irregular Xylem 9-Like); *IRX14L* (Irregular Xylem 14-Like); *IRX7* (Irregular Xylem 7); *DUF579* (Domain Of Unknown Function 579); *GUX* (Glucuronic Acid Substitution Of Xylan); *RWA* (Reduced Wall Acetylation); *INV* (Invertase); *HXK* (Hexokinase); *GGP* (GDP-D-Glucose Pyrophosphorylase); *G6PI* (Glucose-6-Phosphate Isomerase); *M6PI* (Mannose-6-Phosphate Isomerase); *PMM* (Phosphomannomutase); *GMP* (GDP-D-Mannose Pyrophosphorylase); *CSLA3* (Cellulose Synthase-Like A3); and *GGT* (Glucomannan-1;6-Galactosyltransferase). The seasonal wood formation of CW and OW was shown by the up- and down-regulated of each gene in SP compared to FA, indicated as the color bar (blue to black, respectively). The CW formation in SP was shown by the up- and down-regulated of each gene in CW compared to OW, indicated as the color bar (red to green, respectively). The fold change value was the average of two sampling sets. CW (Compression Wood), OW (Opposite Wood), SP (Spring), SM (Summer), and FA (Fall).

**Figure 6:**
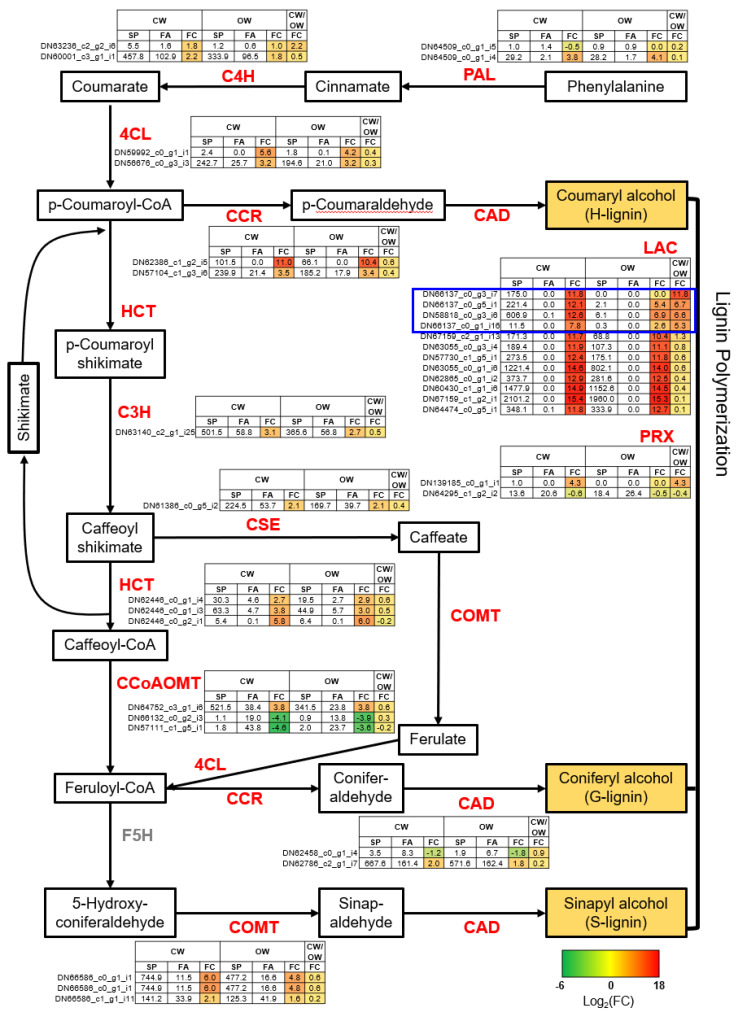
The seasonal transcriptional regulation of critical genes in the lignin biosynthetic pathway in the secondary cell wall formation. Metabolites in each step of the lignin biosynthetic pathway are shown in the box, and all related genes are shown to the side. *PAL* (Phenylalanine Ammonia-Lyase); *C4H* (Cinnamate 4-Hydroxylase); *4CL* (4-Coumarate: CoA Ligase); *CCR* (Cinnamoyl-CoA Reductase); *HCT* (Hydroxycinnamoyl-Coa:Shikimate/Quinate Hydroxycinnamoyltransferase); *C3H* (Coumaric Acid 3-Hydroxylase); *CSE* (Caffeoyl Shikimate Esterase); *COMT* (Caffeic Acid O-Methyltransferase); *CCoAOMT* (Caffeoyl-Coenzyme A 3-O-Methyltransferase); *CAD* (Cinnamyl-Alcohol Dehydrogenase); *LAC* (Laccase); *PRX* (Peroxidase); and *F5H* (Ferulate 5-hydroxylase). The seasonal wood formation of CW and OW was shown by the up- and down-regulated of each gene in SP compared to FA, indicated as the color bar (blue to black, respectively). The CW formation in SP was shown by the up- and down-regulated of each gene in CW compared to OW, indicated as the color bar (red to green, respectively). The fold change value was the average of two sampling sets. CW (Compression Wood), OW (Opposite Wood), SP (Spring), SM (Summer), and FA (Fall).

We aimed to identify key genes involved in the SCW biosynthesis that are critical for CW formation as a response to seasonal changes. For this, we reconstructed the biosynthesis pathways of SCW components (cellulose, galactoglucomannan, xylan, and lignin) using previously reported genes in *P. densiflora* [4] (Figure 5 and Figure 6). With sucrose or phenylalanine as a starting molecule, cellulose/galactoglucomannan/xylan and lignin biosynthetic pathways are shown in Figure 5 and Figure 6, respectively. The enzymes involved in each step of the biosynthetic pathway are encoded by multiple genes, but genes (i.e., contigs) with significant expressions were shown (Figure 5 and Figure 6).

Interestingly, genes such as *INV*, *HXK*, *PGM*, and *UGP* involved in the initial metabolism of sucrose did not significantly increase their expression in spring compared to fall (Figure 5). Only *GGP* and *CSLA3*, responsible for glucomannan production, were upregulated in spring, suggesting seasonal regulation might not significantly impact galactoglucomannan biosynthesis. For cellulose and xylan biosynthesis, all pathway genes were more active in spring than fall, particularly the *CESA* genes (Figure 5). There were no noticeable differences between CW and OW formation (Figure 5).

**Figure 7:**
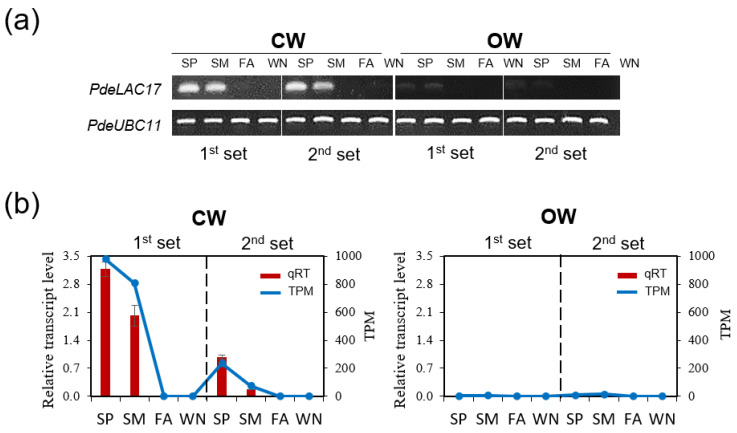
*PdeLAC17* is expressed in CW, specifically. (**a**) Seasonal expression of PdeLAC17 in CW and OW. Semi-quantitative RT-PCR was performed using cDNA prepared from total RNAs extracted from each sample. *PdeUBC11* (DN59720_c0_g1_i20) was used as a loading control. PdeLAC17 (DN58818_c0_g3_i6). (**b**) Validation of *PdeLAC17* expression. In each plot, blue line (right *Y*-axis) represents TPM (Transcript Per Million) values of *PdeLAC17* from RNA-seq data, whereas red bars (left *Y*-axis) show the results of independent qRT-PCR experiments. Error bars indicate SD (n = 3).

For lignin biosynthesis, pathway genes were significantly upregulated in spring (Figure 6). While *PRX* (*Peroxidase*) was reported to be insignificant in gymnosperms [4], our data supported this finding. Some transcripts belonging to *CCoAOMT* were up-regulated in FA may have been due to the response against UV-B [14]. We noted several laccases, responsible for monolignol polymerization, were specifically upregulated in CW compared to OW, with the contig ‘DN58818_c0_g3_i6’, the most significantly CW-specific expressed, which we named *PdeLAC17* due to its similarity to Arabidopsis *LAC17*. This was further validated by both semi-quantitative RT-PCR (Figure 7a) and qRT-PCR (Figure 7b).

SP, spring; SM, summer, FA, fallk WN, winter.

### 3.6. Identifying Differentially Expressed Genes Involved in CW Formation

As genes involved in SCW formation in CW were majorly upregulated from spring to summer, common differentially expressed genes (DEGs) during this period might have been potential candidates for CW formation. There were only a few genes (50 transcripts) that were upregulated from spring to summer (Supplementary Figure S4a). The upregulated genes were involved in lignin biosynthesis, whereas many defense response genes were downregulated in summer (Figure S4b). The common upregulated genes were involved in SCW formation (e.g., *LAC*, *MAT2*, *KAM1*, and *FLA11*), auxin, cytokinin, and strigolactone signaling (e.g., *MES17*, *ARR3*, and *LBO1*), whereas several negative regulators and stress response genes were downregulated in summer, with most related to the ethylene response (Figure S4b, Supplementary Table S4).

**Figure 8:**
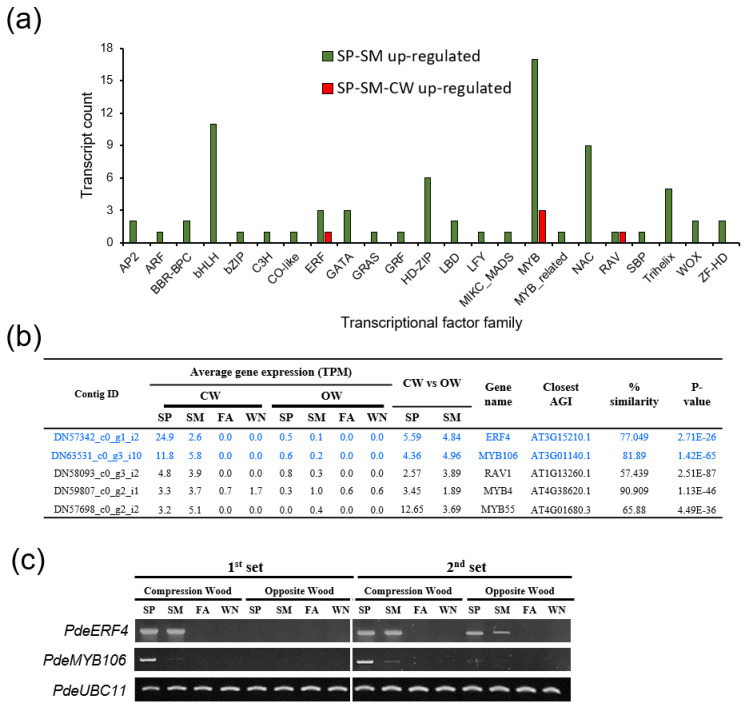
Identification of transcription factors preferentially expressed in CW formation. (**a**) Analysis of transcription factor (TF) family. Number of TFs up-regulated in the growing season (SP and SM) was plotted (green bars). CW up-regulated TFs were shown in red bars. (**b**) Partial list of transcription factors abundantly expressed in CW of growing season. Top two highly expressed TF were marked by blue. (**c**) Seasonal expression of *PdeERF4* and *PdeMYB106* in CW and OW. Semi-quantitative RT-PCR was performed using cDNA prepared from total RNAs extracted from each sample. PdeUBC11 (DN59720_c0_g1_i20) was used as a loading control. CW (compression wood), OW (opposite wood), SP (Spring). SM (Summer), FA (Fall), and WN (Winter). AGI: Closest Arabidopsis Gene Index.

Subsequently, we sought to identify transcription factors (TFs) upregulated during CW formation (Figure 8, Supplementary Table S5). Among the many TF families found to be upregulated from spring to summer, we identified TFs significantly upregulated in CW in both spring and summer (PdeERF4 and PdeMYB106) (Figure 8a,b). Their expression patterns were confirmed by semi-quantitative RT-PCR (Figure 8c).

### 3.7. Functional Examination of PdeERF4 and PdeMYB106 as Possible Regulators of Laccase Activity

We speculated that the TFs highly expressed in CW might regulate PdeLAC17. As laccases have an oxidative function, they are known to oxidize the biphenolic dye ABTS to a stable cationic radical state [47].

**Figure 9:**
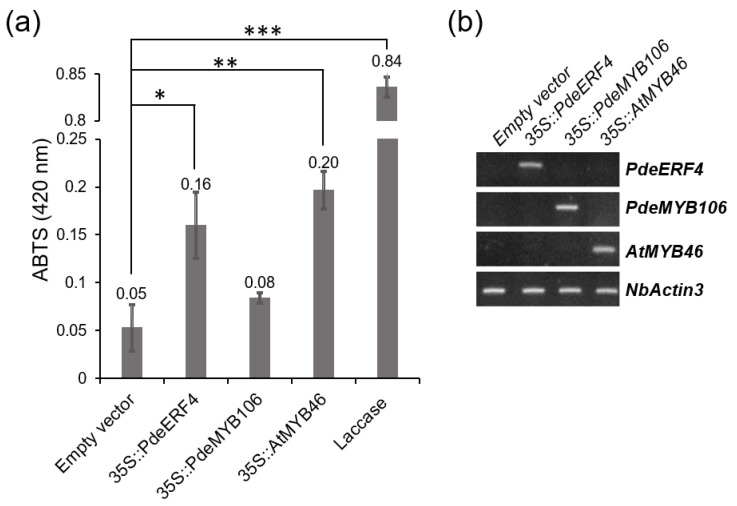
Laccase activity measurement of transient overexpression of *PdeMYB106* and *PdeERF4* in tobacco leaf. (**a**) ABTS assay using crude protein extracted in infiltrated leaf area. Laccase 500 ng/μL was used as a positive control for this assay. *35S::AtMYB46* was used as infiltration positive control. Error bars indicate SD (n = 3). Student *t*-test, * (*p* < 0.05), ** (*p* < 0.01), and *** (*p* < 0.001). (**b**) Semi-quantitative RT-PCR to confirm the expression of transgenes in tobacco leaf. cDNAs prepared from total RNAs extracted from each sample were used as PCR templates.

For the preliminary characterization of PdeERF4 and PdeMYB106 functions, we transiently introduced 35S::PdeERF4 or 35S::PdeMYB106 constructs into tobacco leaves and assessed the oxidative activity after 4 days of infiltration (Figure 9). 35S::AtMYB46, a known TF regulating AtLAC17 [5], was used as a positive control and showed a substantial increase in ABTS absorbance (Figure 9a). 35S::PdeERF4 significantly boosted oxidation, whereas 35S::PdeMYB106 showed no significant effect (Figure 9a). The expressions of the introduced transgenes were confirmed by semi-quantitative RT-PCR (Figure 9b). These findings suggest that PdeERF4 might positively influence the activity of laccases.

## 4. Discussion

### 4.1. Seasonal Variation in Gene Expression in the CW and OW Formation

During the growing season, the majority of genes involved in wood formation in *P. densiflora* were activated, whereas those linked to defense or stress response were suppressed (Figure 3 and Figure S2). The decline in expression of most SCW biosynthetic genes post-summer illustrated a seasonal trend in the activation of these genes. Strikingly, genes like Cellulose Synthase (CESAs) and Laccases (LACs) were not detected in the fall and winter (FA) (Figure 4 and Figure S1). A few lignin biosynthetic genes such as *PdeC4H* and *PdeCOMT* remained active but at reduced levels during FA and WN, possibly providing cold and UV radiation protection (Figure 4 and Figure S1) [49].

The transcriptomic profile differed significantly between the active growth (spring—SP) and late growth (summer—SM) stages of the growing season (Figure 2b). Spring vs. fall exhibited more DEGs than summer vs. fall (Supplementary Figure S2). SP activated genes implicated in cell wall formation, cell division, and differentiation. On the other hand, SM triggered functions associated with growth suppression, such as senescence and defense response (Figure 3, Supplementary Figure S2).

Wood formation in CW was notably increased from SP to SM, whereas, in OW, stress response genes were activated during SM. Previous research on mountain pine (*Pinus mugo* Turra ssp. Mugo) revealed that CW formation took longer than OW formation, with CW cells spending more time in the wall-thickening phase [50]. Principal component analysis (PCA) showed significant differences in wood formation between CW and OW during SM (Figure 2b). During SP, CW and OW showed a similar number of up-regulated genes associated with hormone regulation, cell division and expansion, SCW formation, and programmed cell death (PCD). However, OW had a significantly higher number of up-regulated genes than CW in SM, primarily related to defense and stress response (Figure 3, Supplementary Figure S2). This suggests that OW may face more environmental stress or attacks than CW during SM.

Both CW and OW showed distinct responses to changes in weather conditions, specifically precipitation. While previous studies have suggested that high precipitation leads to larger xylem cell size [51,52], our results showed that precipitation did not affect gene expression related to cell division and expansion (Figure 4). Interestingly, high precipitation in the first set of SPs coincided with strong induction of SCW formation genes in both CW and OW. But, under the lower precipitation conditions in the second set, CW maintained high gene expression, whereas OW’s expression decreased (Figure 1c and Figure 4).

Taken together, CW and OW may respond differently to seasonal shifts and weather changes. CW experiences increased wood formation from SP to SM, whereas it decreases in OW during the SM phase, prioritizing the defense response. The formation of SCW and PCD in OW seems to coincide with precipitation levels, whereas CW appears to be independent of these conditions.

### 4.2. Hormonal Regulation of Wood Formation in Seasonal Change

Auxin promotes cell proliferation and growth, and cytokinin stimulates cambium reactivation and division, whereas GA fosters cell differentiation and elongation [8]. The genes related to auxin and cytokinin signaling were strongly activated in SP but less so in SM (Figure 3). Some genes like MES17 (involved in auxin biosynthesis) [53] and ARR3 (implicated in cytokinin response and circadian clock regulation) [54] showed an up-regulation in CW (Table S4). SPY, a dual-function regulator negatively affecting GA while enhancing cytokinin and contributing to circadian clock regulation [55], showed reduced expression in both SP and SM (Figure 3). MYC2, a gene that activates JA response genes, was specifically up-regulated in OW-SM, which aligns with JA’s reported role in stress response and reduced presence in CW of gymnosperm [13,56,57].

Ethylene, despite some conflicting reports, might also increase CW formation and stimulate cambial growth in tension wood [8,13,58,59]. In our study, ethylene response factors, such as ERF-1, ERF12, and ERF2, showed decreased activity in both CW and OW during the growing season. Interestingly, *PdeERF4*, induced by ethylene, JA, and abscisic acid [60], was highly expressed in CW (Figure 8). EDF3 and ERF110, which are involved in flower senescence and flowering time, respectively [61,62], were down-regulated in both SP and SM and were specifically induced in OW SM (Figure 3, Supplementary Table S4).

In summary, auxin, cytokinin, and GA seem to positively regulate wood formation, whereas JA seems to act as a negative regulator. Ethylene’s role appears to be complex, promoting both senescence and CW formation.

### 4.3. Identifying Key Genes in Compression Wood Formation in P. densiflora

Following the identification of critical genes in lignin, cellulose, and hemicellulose biosynthesis in *P. densiflora* as previously reported [4], we were able to pinpoint those involved in SCW formation. We found that their expression patterns correlated with seasonal changes and were more abundantly expressed in CW (Figure 5 and Figure 6). The activities of these genes predominantly increased during the SP and decreased in FA. In the context of previous research, it was noted that CW had an increase in not only lignin-related genes (such as PAL, C3H, 4CL, CAD, HCT, and CCoAOMT) but also those related to cellulose biosynthesis (such as CSLA) [21,24]. In contrast, our study observed no abundant expression in CW of these genes, with the exception of laccase (Figure 5 and Figure 6).

The laccase gene PdeLAC17, important for monolignol polymerization [63], was consistently upregulated in CW (Figure 6, Table S4). Within the identified laccase transcripts, some showed CW abundance, whereas others did not (Figure 6, Supplementary Table S4). This aligned with findings from other conifer studies, suggesting a differential regulation of laccase family members during CW development [22,64,65]. Among the 50 transcripts up-regulated in CW, we identified two transcription factors—PdeERF4 and PdeMYB106—as the most significantly upregulated (Figure 8, Supplementary Figure S4). Interestingly, 35S::PdeERF4 exhibited significant oxidation activity in tobacco leaf, similar to 35S::AtMYB46 (Figure 9 and Figure S5). This suggested that PdeERF4 may contribute to the induction of laccase activity in CW.

## 5. Conclusions

Our research has provided a comprehensive view of the intricate dynamics of gene expression involved in the wood formation of *P. densiflora*. The analysis distinctly revealed seasonal shifts in the activation of genes related to wood formation and stress responses. The CW and OW displayed differing responses to such shifts and weather conditions, demonstrating the complex relationship between environmental factors and genetic regulations of wood formation. A range of genes contributing to SCW formation were identified, with varying levels of expression across seasons and weather conditions. Notably, the laccase family member *PdeLAC17*, associated with monolignol polymerization, showed consistent upregulation in CW. Among the transcripts upregulated in CW, we identified two transcription factors that were significantly upregulated, PdeERF4 and PdeMYB106, potentially contributing to laccase induction in CW. Taken together, our findings underscore the importance of a multifaceted approach, incorporating environmental, hormonal, and genetic factors, to comprehensively understand the regulation of wood formation. This study contributes to our understanding of the complex processes governing wood formation and presents potential gene targets for enhancing wood production and quality in forestry and bioenergy industries. Further research is required to explore the full complexity of these interactions and to validate the role of the identified genes and their possible applications.

## Supplementary Materials

The following supporting information can be downloaded at: https://www.mdpi.com/article/10.3390/genes14091698/s1, Figure S1: Verification of the sampling by expressions of marker genes, Figure S2: Characteristics of DEGs in CW and OW by seasonal changes, Figure S3: Expressional changes in significant transcript involved in the wood formation (CW and OW) over four seasons in two consecutive years, Figure S4: Differentially expressed genes (DEGs) categorization of the CW in the growth season (SP and SM), Figure S5: Coding sequences of *PdeERF4* and *PdeMYB106*, Table S1: Temperature and day length in the sampling days, Table S2: Summary of RNA-seq analysis, Table S3: The expression of well-known genes involved in wood formation of CW and OW in growing season, Table S4: List of typical compression wood up- and down- regulated genes, Table S5: List of typical compression wood up- and down- regulated transcriptional factors, Table S6: Primer sequences used in this study.
