# Supplementary material for: Seasonal Developing Xylem Transcriptome Analysis of Pinus densiflora Unveils Novel Insights for Compression Wood Formation
Source: Genes (Basel). 2023 Aug 26;14(9):1698. doi: 10.3390/genes14091698 (PMC10531420; doi:10.3390/genes14091698)
Supplement: Supplementary file 1 [file genes-14-01698-s001.zip › genes-2557687 supplementary.pdf]

Supplemental Figure S1. Verification of the sampling by expressions of marker genes.

Semi-quantitative RT-PCR was performed using cDNA prepared from total RNAs extracted from each sample. The marker genes of *P. densiflora* (e.g., *PdeNAC2*, *PdeMYB46*, and so on) are homologous to Arabidopsis genes involved in the transcriptional regulator, cellulose biosynthesis, lignin monomer biosynthesis and lignin polymerization. And *PdeUBI* was used as a loading control.

Supplemental Figure S2. Characteristics of DEGs in CW and OW by seasonal changes.

(a) Identification of DEGs both up or down-regulated in CW and OW. DEGs were found by comparison of Spring and Fall or Summer and Fall, respectively. (b) Venn diagram shows the number of DEGs in CW vs. OW. in relation to season. (c) Gene Orthology (GO) analysis for Biological Process of up- or down-regulated genes in (a). Top significance GO terms was plotted with the negative logarithmic adjusted p-value of enrichment. CW (Compression Wood), OW (Opposite Wood), SP (Spring), SM (Summer), FA (Fall).

Supplementary Figure S3. Expressional changes of significant transcript involved in the wood formation (CW and OW) over four seasons in two consecutive years.

All the significantly expressed transcripts of the genes involved in cellulose, hemicellulose, lignin biosynthesis, and PCD which described in Figure 4 were plotted to confirm the differences in CW and OW for two years.

Supplementary Figure S4. Differentially expressed genes (DEGs) categorization of the CW in the growth season (SP and SM).

(a) Venn diagram shows the number of DEGs in growth season (SP and SM). (b) Gene Orthology (GO) analysis for Biological Process of SP, SM specific and SP-SM commonly expressed DEGs which classified in (a). Top significance GO terms was plotted with the negative logarithmic adjusted p-value of enrichment analysis. CW (Compression Wood), OW (Opposite Wood), SP (Spring), SM (Summer), FA (Fall).

Supplementary Figure S5. Coding sequences of *Pde ERF4* and *PdeMYB106*

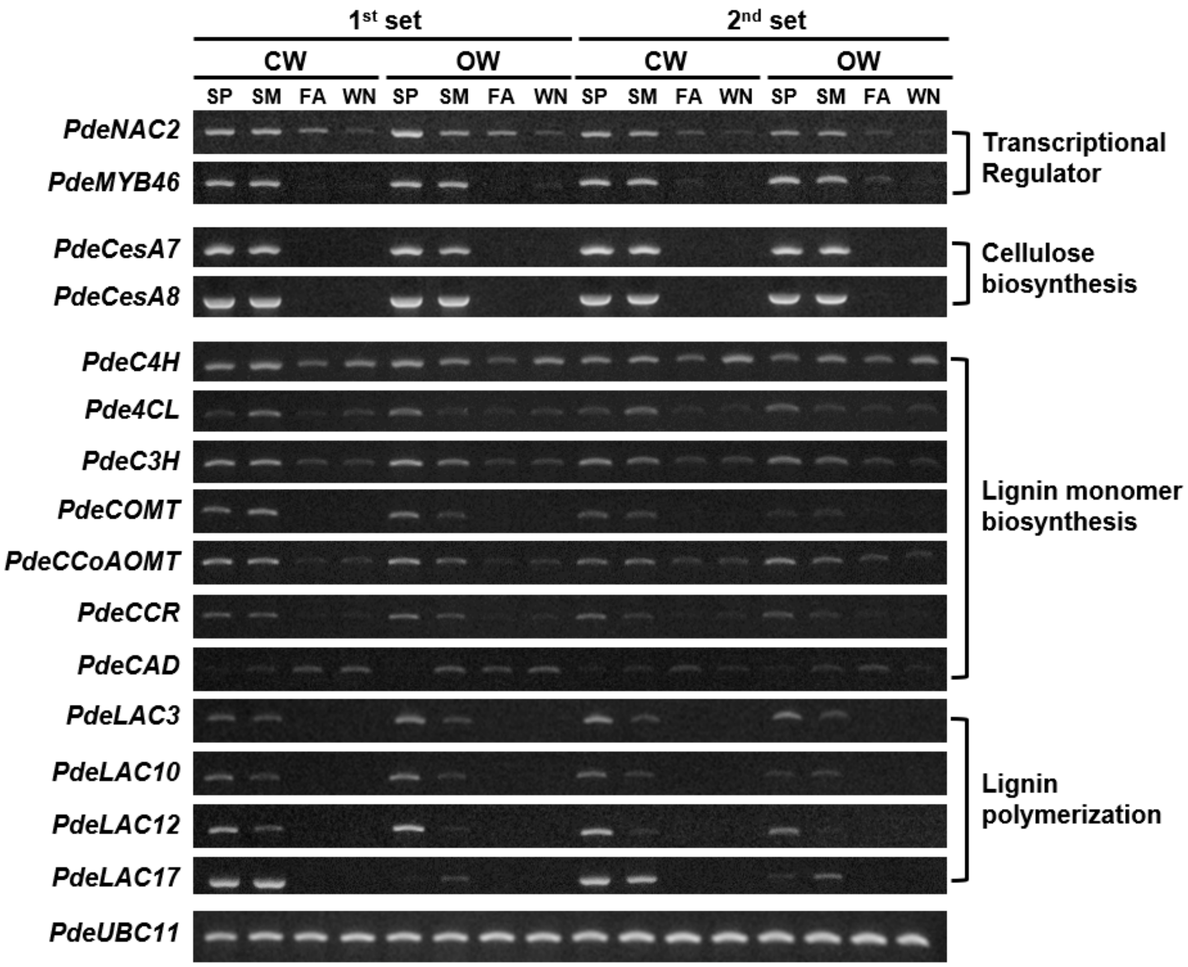

Supplemental Figure S1. Verification of the sampling by expressions of marker genes.

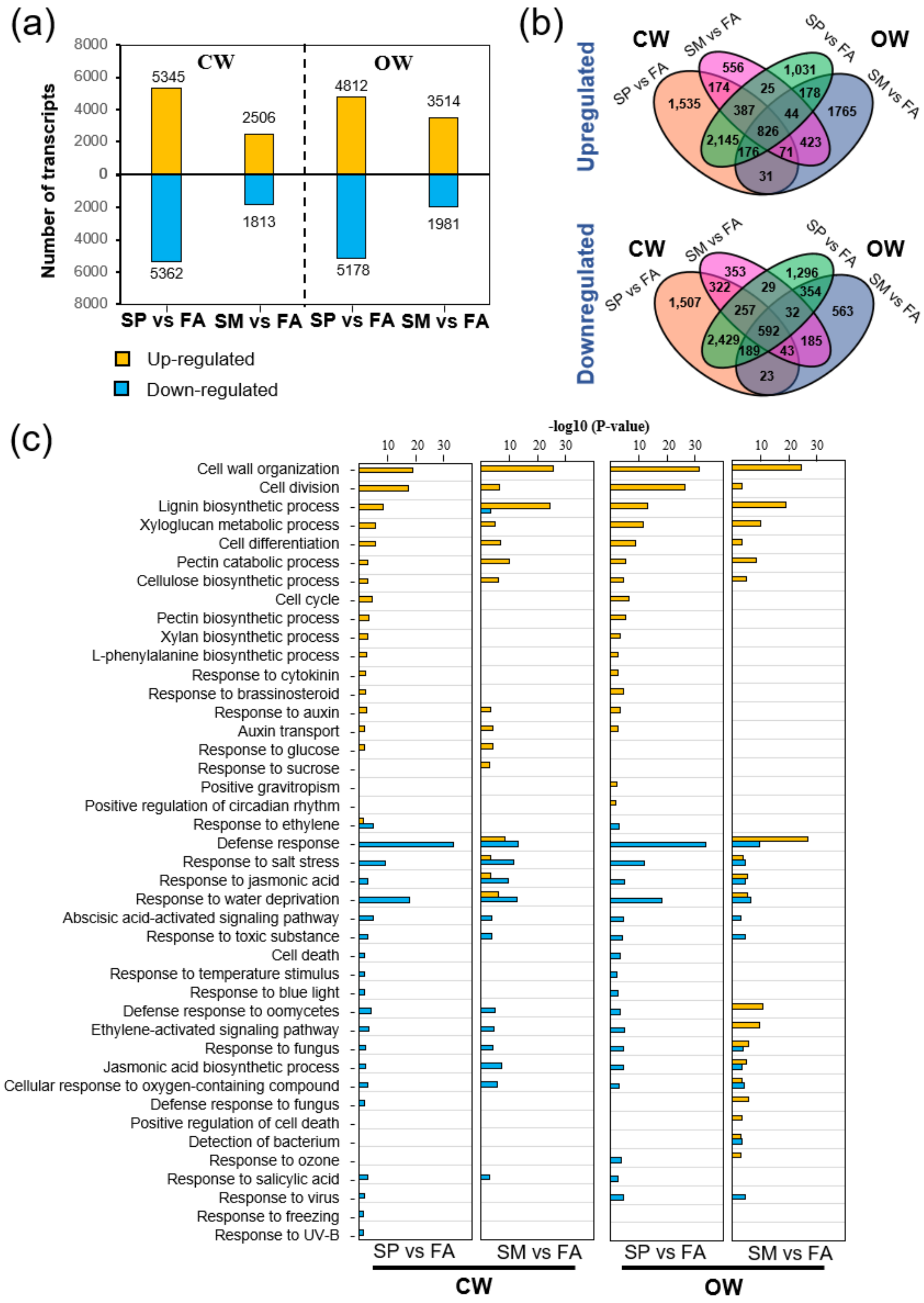

Supplemental Figure S2. Characteristics of DEGs in CW and OW by seasonal changes.

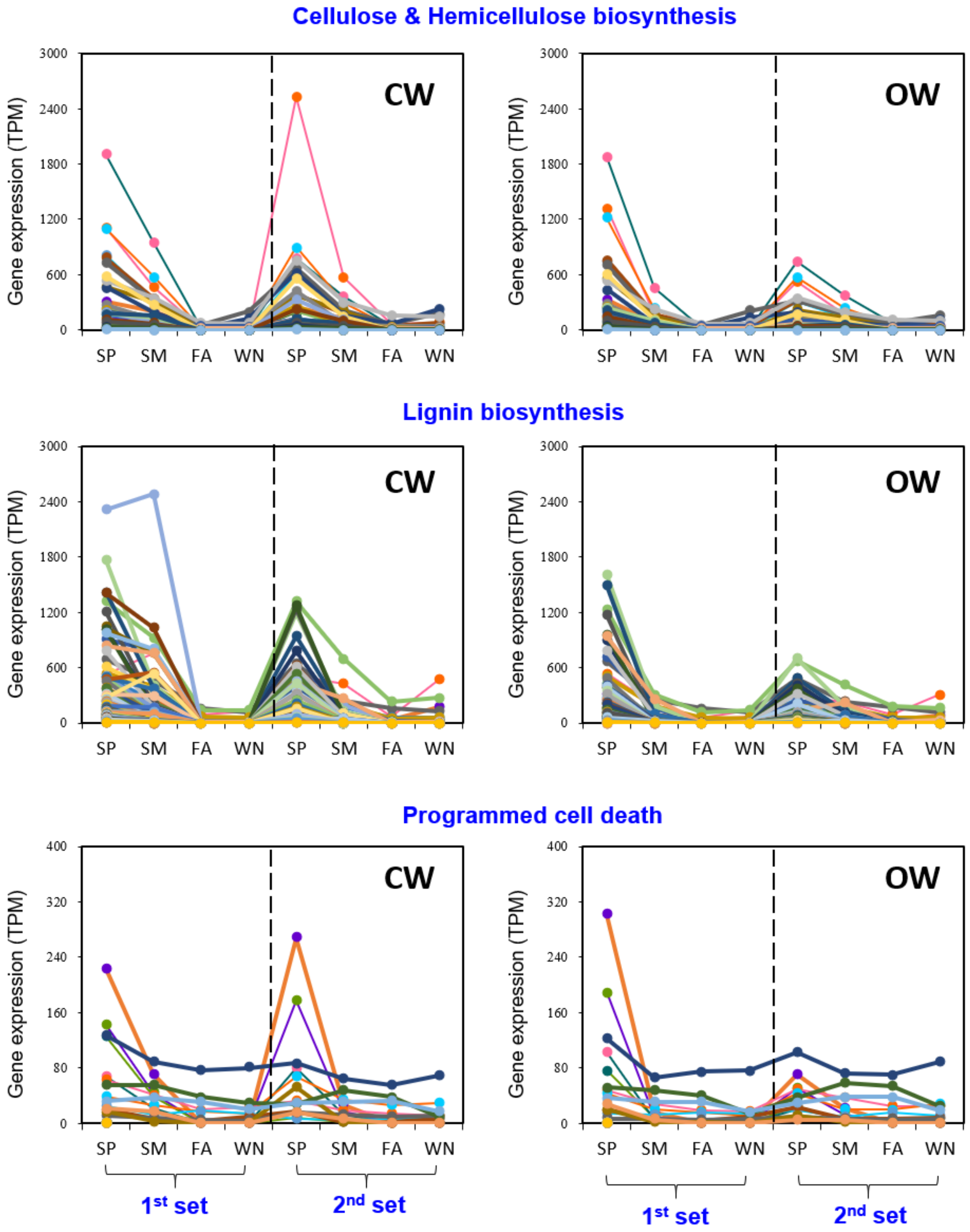

Supplementary Figure S3. Expressional changes of genes involved in the wood formation (CW and OW) over four seasons in two consecutive years.

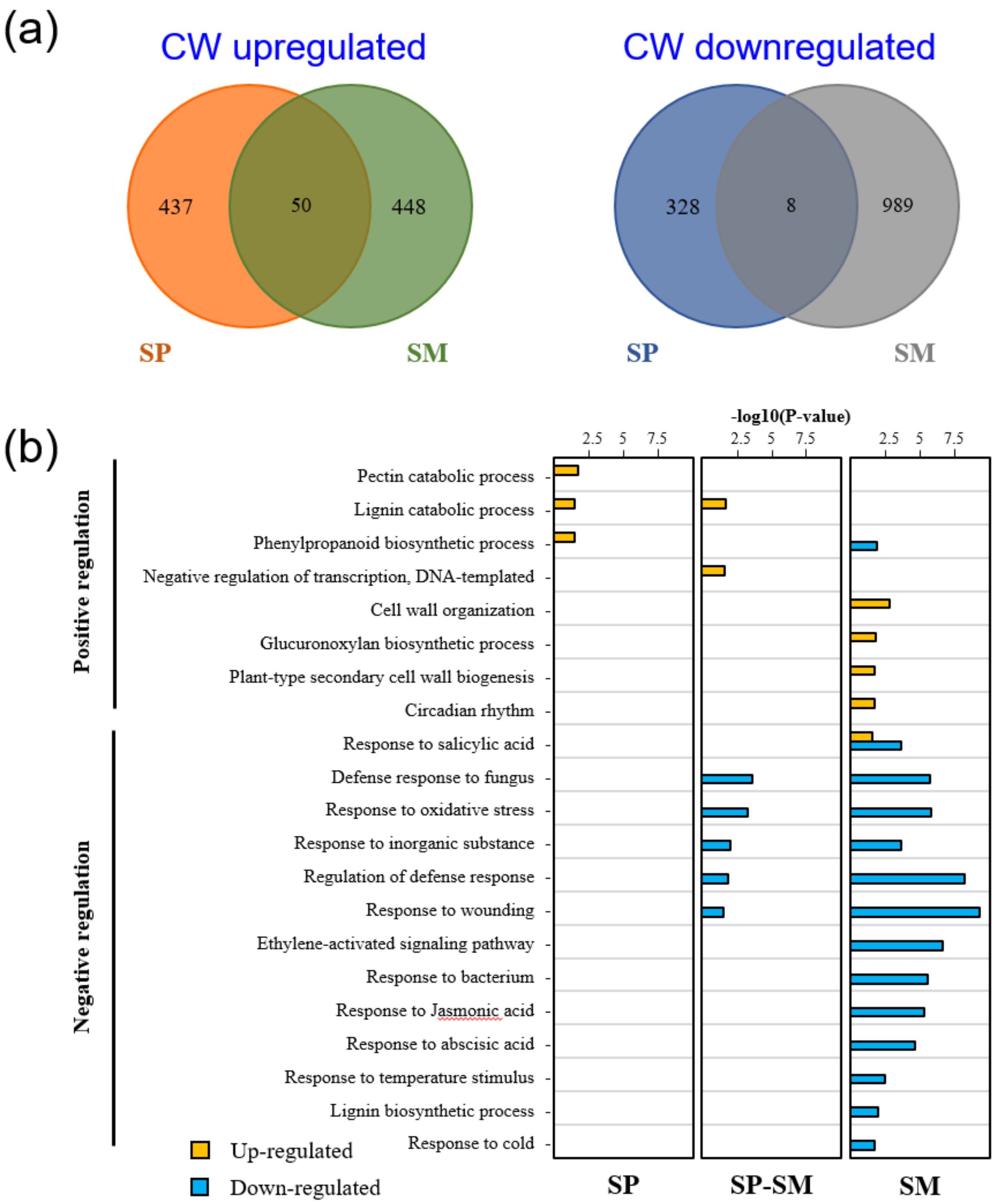

Supplementary Figure S4. Histogram of differentially expressed genes (DEGs) categorization of the CW in the growth season (SP and SM).

>PdeERF4_ DN57342_c0_g1_i2 _813bp

ATGGCGGCTCACAGACATAGAAATGGGGATGGGCATAGCGAGATCCTCGGCGATTGTGATGCTCATG

TGGTTAAAGAAGTGCATTATAGAGGCGTCAGGAGGAGGCCTTGGGGTCGATATGCAGCAGAGATCAC

AGACCCATTAACGAAAAAACGTAAGTGGCTGGGCACTTTCGACACAGGCGAGGAGGCAGCCAGGGC

CTATGACAAAGTCGCCAGAAATATGAGAGGAGCCAACGCCAAAACCAACTTCCCGGCTGCCTCCGTC

CAGAGCGAAGCCTTCGGCATCCAGAGCGGCACGATCTCCATTGCTACCAGTCTTCCAGATCCTCCGCT

TTCCTCTACGTCTGCGAATGATTGTAAAATAAAAAACAAGGTGTCTGCCAGTTTCGGCGTAGCTAAAC

AAGGGAAACCATGCACGGCCATTCACGTGAATGAGGGAGTGGGCGATGACAACATGAATCGCACTGC

TACTGCCATTCTGGACTACGCTACTCTTCTGGAGAAGCTACCTATGGGGGTGTCGTTGTTAGGGGAGAT

TGTGGGATCTCAGATGATCAACAGACACGGAGGCCATGATTGGATGGGAGTAGGGTTAGTGAACAAT

TTGCTTACTGCAACCCCGAAGTCAAGCGTTTGCGATTCACCTTCATCTTCGGGCTTCGTTGACACACAA

CTCTCTCCTCCACCTCCTGCAAACGAAAACGCCACGCTGCAGTCAATCGATTTGAATGAAGATGAGCG

TTCCCCGTCTCTGACTCTTTCTCTTGGATACGATTTTCTGAACAAATTCTGTGTGGGATACAAGGATTAG

>PdeMYB106_ DN63531_c0_g3_i10_985bp

ATGGGTAGGGCTCCCTGCTGCGAAAAGGTTGGGCTCAAGAAGGGCCCCTGGACGCCGGAGGAAGATC

AAAAGCTCCTCGCTTACATACAGGAGCACGGCCATGGCAGCTGGAGGGCTCTGCCTCAGAAAGCTGG

GTTGCTGAGATGCGGGAAAAGCTGCAGATTGCGTTGGGCTAACTATCTAAGGCCAGATATCAAGCGG

GGAAAGTTCAGCACACAGGAAGAACAGACGATTATTCAACTTCATGCCCTTCTGGGAAACAGGTGGT

CCACCATCGCCACTCACCTTCCTAAGCGAACTGACAACGAAATCAAGAACTACTGGAATACCCATCTG

AAAAAGCGCTTGGTGCAGATGGGAATCGACCCCATGACGCACAAGCCGAAGTCCGAATCGACTATTG

CAGTTGGCCTTCAGTCGCGTAATGGGTCGTCGAATCTGAGCCATATGGCGCAGTGGGAGAGCGCGCGC

CTGGAAGCCGAATCGAGGTTGGCGCGCGAGTCCAAGCTCAGGGCCCAGGGCCTATGGCCTGGGTCCA

TGAGAAACCCTAATGTGAACTCAGATATACTTACTAGATTGCGGTTAGCCAGTCAAGGATTCGATTCC

GATAAGGAAGGGTCAAAGATTCTGGGGTTGAACTCTACTGGCGGCTTTGAAGGTATGAAAATAGCAG

ACAAATGGGACAAGTTCCAAGATTTGAGTTTGGGGATTAAGTCAGACCAAAGCAATTCTTCTGCGCTG

GGAAGTTCCTTCAGAGGTTATAATCCACTGGAGAATGTTCATAGCGCGGACCGAGCCAGTAACACAA

GTTATCTCAGCCTTCTGCAACAAGCCATGTGTCCTTCTGCTCAAGGCAGCAAAATTCTGTACAATGAGG

CCAAGAAGCTGATGGAGAACCAGAAGAATCGGCAGGATAATGTAACGGCCACCCTTTATAATGTCCA

CCAACCTCATGGAAAATTGCAGTGAAAGCAAGCATCTATGA

Supplementary Figure S5. Coding sequences of *Pde ERF4* and *PdeMYB106*
